# Magnetic resonance imaging-negative myeloneuropathy and bilateral facial paresis unfurling systemic lupus erythematosus

**DOI:** 10.5339/qmj.2022.42

**Published:** 2022-09-12

**Authors:** Ritwik Ghosh, Shambaditya Das, Koustav De, Souvik Dubey, Julián Benito-León

**Affiliations:** ^1^Department of General Medicine, Burdwan Medical College and Hospital, Burdwan, West Bengal, India; ^2^Department of Neuromedicine, Bangur Institute of Neurosciences, Institute of Post Graduate Medical Education and Research & SSKM Hospital, Kolkata, West Bengal, India; ^3^Department of Neurology, University Hospital “12 de Octubre,” Madrid, Spain; ^4^Centro de Investigación Biomédica en Red Sobre Enfermedades Neurodegenerativas (CIBERNED), Madrid, Spain; ^5^Department of Medicine, Complutense University, Madrid, Spain

**Keywords:** MRI-negative myeloneuropathy, neuropsychiatric, lupus erythematosus, myelopathy, bifacial-facial-paresis, rituximab

## Abstract

Systemic lupus erythematosus is a chronic autoimmune connective tissue disorder that can affect all the neuroaxes in the central and peripheral nervous systems. Myelopathy in systemic lupus erythematosus is one of the least common neuropsychiatric syndromes accounting for 1%–2% of cases. Myelopathy has long been diagnosed based on clinical findings, laboratory tests, and gold-standard gadolinium-enhanced magnetic resonance imaging (MRI). MRI-negative myelopathy is a recently described subset of myelopathies. Here, we report the case of a young woman from rural West Bengal, India, who presented with overlapping features of white-matter and gray-matter myelopathy associated with peripheral neuropathy and bilateral asymmetric lower motor neuron-type facial paresis. The historical analysis yielded clues toward an etiological diagnosis of systemic lupus erythematosus, further substantiated by seropositivity of lupus-specific autoantibodies. Her neurological disabilities responded poorly to oral administration of hydroxychloroquine, bolus intravenous administration of methylprednisolone, and high-dose cyclophosphamide therapy but eventually responded remarkably well to cyclical rituximab therapy. This case adds to the tally of cases of MRI-negative lupus myelopathy. MRI-negative myelopathy in systemic lupus erythematosus can be easily missed if not meticulous attention is paid during clinical history taking and examinations.

## Introduction

Systemic lupus erythematosus (SLE) is a chronic autoimmune connective tissue disorder that can affect all the neuroaxes in the central and peripheral nervous systems in various combinations.^1^ SLE-associated myelopathy is one of the least common neuropsychiatric syndromes, accounting for 1%–2% of cases.^2^ It is often difficult to diagnose and manage, portending a poor prognosis.^2^ The diagnostic dilemmas are further accentuated when a clinically suspected myelopathy is not supported by imaging evidence on spinal cord magnetic resonance imaging (MRI). MRI-negative SLE myelitis has rarely been reported.^3^

Herein, we present a novel case of SLE unmasked by MRI-negative myeloneuropathy and bilateral lower motor neuron-type facial paresis.

## Case Report

A 26-year-old previous healthy woman from rural West Bengal, India, was brought to the emergency department for complaints of sudden-onset urinary retention, which relieved by catheterization, followed by sequential weakness of all four limbs, which developed within 6–8 h, for the last 5 days. She also complained of deviation of her mouth’s angle toward the left side, drooling of saliva and food particles, inability to close her right eye, decreased taste sensation, and difficulties in whistling and blowing for the same duration. She had no history of associated or preceding febrile episode, loss of consciousness, seizures, headache, vomiting, respiratory distress, localized/radiating neck pain, girdle-like sensation, muscle pain, stiffness, joint pains, or recent travel. In addition, her family had no history of similar ailments. Detailed probing revealed a history of frequent delayed-healing oral ulcers and photosensitivity for the last 18 months, which started after her first childbirth. She had a bad obstetric history regarding unevaluated recurrent pregnancy loss (four episodes; all occurred in mid-trimesters) for the last 3–4 years preceding this first uneventful live childbirth.

A general survey revealed pallor, oral ulcers, malar rash, photosensitivity, noncicatricial alopecia, and mild splenomegaly. Detailed neurological examination revealed intact cognitive functions, asymmetric bilateral lower motor neuron-type facial paresis (right more than left), reduced taste sensation on both sides of the anterior tongue, and hyperacusis. She also had asymmetric quadriparesis involving all four limbs (Medical Research Council grading, upper limbs (bilateral): proximally 4 − /5, distally 3+/5; lower limbs: proximally 2/5 and 3/5, distally 1/5 and 2/5 on the right and left sides, respectively). She had generalized hypertonia and hyperreflexia, except around the ankle joints, which on examination were found to be areflexic and hypotonic with extensor plantar responses (i.e., positive Babinski’s sign). Sensory examination revealed reduced pinprick sensations and decreased joint position and vibration senses up to midshin levels. Cerebellar functions and gait could not be accurately evaluated. The examination results of the bony cranium and spine were normal. No signs of meningeal irritation or intracranial hypertension were observed.

**Figure 1.**
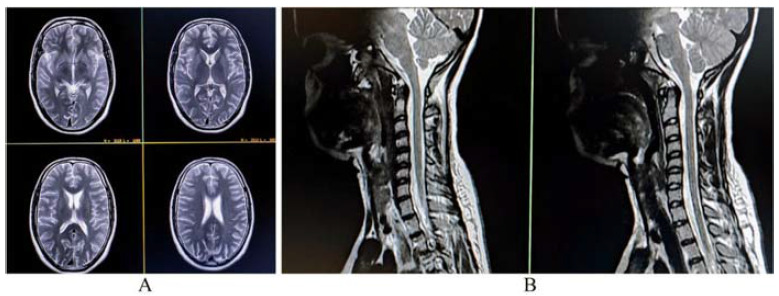
Magnetic resonance imaging of the brain revealing no abnormalities on axial tbl2-weighted imaging (A). Sagittal tbl2-weighted imaging of the cervical spine also was normal (B).

A complete blood cell count revealed anemia and thrombocytopenia (hemoglobin 8.0 g/dL, platelet counts of 90000/µL), with increased erythrocyte sedimentation rate (ESR; 66 in the first hour) and increased lactate dehydrogenase level (LDH; 1256 U/L; reference range < 248.00). The blood bank reported difficulties in performing compatibility testing and cross-matching. Direct Coomb’s test was positive (suggestive of autoimmune hemolytic anemia; later confirmed to be IgG and warm type). Liver function tests revealed unconjugated hyperbilirubinemia (unconjugated bilirubin of 3.2 mg/dL, reference range < 1.1) with mild transaminitis (aspartate transaminase 70 U/L; alanine aminotransferase of of 76 U/L). Thyroid (including antithyroid antibodies), kidney function tests and glycemic indices were within normal ranges. Urinalysis was normal. Serum electrolytes, arterial blood gases, muscle enzymes, and C-reactive protein all were within normal range. Gadolinium-contrasted MRI of the brain, orbits, and spinal cord did not detect intra- or extra-axial lesions (Figure 1 ).

On day 5 of hospitalization, a nerve conduction study, coupled with needle electromyography, revealed a sensorimotor (motor more than sensory) polyneuropathy (predominantly axonal) affecting the lower limbs. The cerebrospinal fluid (CSF) study revealed lymphocytic pleocytosis, with raised protein levels and low glucose levels (white blood cell count, 30/μL; protein, 106 mg/dL; glucose, 43 mg/dL; corresponding capillary blood glucose, 112 mg/dL; oligoclonal bands, negative). Relevant neuroviruses (adenovirus, enteroviruses, Epstein Barr virus, human herpesvirus 6 and 7, human parechovirus, Japanese Encephalitis virus, varicella-zoster virus, cytomegalovirus, and herpes simplex virus types 1 and 2) were tested by polymerase-chain-reaction (qualitative) method and were negative. Relevant tests also excluded dengue, HIV (1, 2) and hepatitis A, B, C, and E. Serum and CSF antiaquaporin 4-antibodies and antimyelin oligodendrocyte glycoprotein antibodies were negative. The visually evoked potential test was normal.

Causes of acute- to subacute-onset myeloneuropathies were searched (Table 1). Nutritional causes were ruled out, as the patient had normal serum B12, folic acid, homocysteine, methylmalonic acid, vitamin E, and copper levels. Metabolic causes were ruled out, as she was euglycemic and non-azotemic, and the urine sample tested for total (qualitative and quantitative) porphyrins and toxins were negative. Drugs and toxins were reasonably ruled out, as she never had any relevant exposure to cassava products, Khesari products, organophosphorus compounds, heavy metals, or hexacarbon compounds, and she had no addictions. Thus, we were left with either infectious, immune-mediated, or paraneoplastic neuropathies. Relevant tests for Borrelia sp., *Campylobacter jejuni, Hemophilus influenzae, Tropheryma whipplei,* and tuberculosis were negative. The oropharyngeal swab test for SARS-CoV-2 by qualitative real-time reverse-transcriptase polymerase-chain-reaction assay was negative. The 18-Fluoro-deoxy-glucose positron emission tomography of the whole body could not find any neoplastic lesion or suspicious lymphadenopathy. Serum protein electrophoresis with immunofixation failed to reveal paraproteinemia. Anti-neuronal (paraneoplastic) autoantibodies profiles were tested and came negative. Critical illness polyneuropathy was ruled out from the historical analysis. Antinuclear antibodies (ANA; Hep-2 method) revealed seropositivity (1:80 titer dilution; homogenous pattern in immunofluorescence). ANA profile (line immunoassay; LIA method) further revealed anti ds-DNA, anti-Sm, and Ribosomal-P-protein seropositivities. Anti-SSA/Ro (52 and 60) and anti-SS-B/La antibodies were negative. Serum angiotensin-converting enzyme and calcium levels were normal, and c-ANCA and p-ANCA were negative. Interestingly, tests for antiphospholipid antibodies yielded positive results for anti-β2-glycoprotein-IgG. Serum C3 and C4 complement levels were low (C3 of 40 mg/dL; reference range, 90–180; C4 of 4.5; reference range, 10–40). Schirmer’s test and a lip biopsy of minor salivary glands yielded negative results, excluding Sjögren syndrome associated with myeloneuropathy. Echocardiography and abdominal ultrasonography revealed mild pericardial and pleural effusion with splenomegaly. The 2019 EULAR/ACR classification criteria then confirmed diagnosis of SLE: constitutional domain, 00; hematologic domain, thrombocytopenia and autoimmune hemolysis score, 04; neuropsychiatric domain, 00; cutaneous domain, non-scarring alopecia and oral ulcers, 02; serositis domain: pleural/pericardial effusion, 05; arthritis domain, 00; renal domain, 00; antiphospholipid antibodies domain, 02; complement protein domain: low C3 and low C4 levels, 04; highly SLE-specific antibodies domain: anti-Sm and anti-ds-DNA antibodies were positive, 06. The total score was 23. Here within each domain, only the highest weighted criterion should be counted toward the total score.^1^

Upon confirmation of autoimmune hemolytic anemia, in a background of SLE, she was immediately put on oral hydroxychloroquine 400 mg/day and high-dose pulse intravenous methylprednisolone therapy (1 g/day for five consecutive days). Although the hemolysis settled with glucocorticoids, no clinically demonstrable neurological improvement was found even after 7 days. Intravenous methylprednisolone therapy was followed by intravenous high-dose cyclophosphamide infusion (1 g once a month) with MESNA. However, after two cycles of intravenous high-dose cyclophosphamide infusion, although the disease severity scores had decreased significantly, neurological improvements were minimal (i.e., return of the ankle-deep tendon reflexes and the partial return of bladder control). She was then put on rituximab infusion therapy (1 g once on days 0 and 14 constitute a single cycle; one cycle can be repeated every 6 months or earlier if necessary). Surprisingly, just after completing the first cycle of rituximab infusion, she showed significant neurological improvement (in terms of increased motor strength, decreased sensory symptoms, and a further improvement in bladder continence and facial weakness). She completed three cycles of rituximab infusions without any drug-related adverse reactions. In the 19th month of follow-up, she had only persisting brisk deep tendon reflexes as the only neurological deficit. Repeated neuroimaging every 6 months until this follow-up failed to reveal any intra- or extra-axial signal changes.

## Discussion

SLE-associated myelopathy, albeit rare, is dreadful and often the presenting manifestation of SLE. It is extremely difficult to diagnose, especially without radiological signs (i.e., MRI-negative myelopathy).^2-6^ In addition, SLE-associated myelopathy can precedeother organ involvements.^2,3,5-7^ In our patient, myelopathy was the presenting manifestation, which led to SLE diagnosis; however, recurrent pregnancy loss was probably the initial manifestation.

According to existing literature, common etiologies of MRI-negative myelopathy include paraneoplastic myelopathy, demyelinating diseases, glial fibrillary acidic protein-related disorders, anti-GAD-65, antiglycine receptor-associated myelopathy, viral myelitis, idiopathic transverse myelitis, and rarely vitamin B12 deficiency myeloneuropathy.^6,8-10^ The timing of imaging (either too early or too late in the disease course) and lesser sensitivity of conventional spinal MRI have been long considered responsible for missing the subtle signal changes (intra- or extra-axial) in these cases by many researchers.^3,6,9,10^ Nevertheless, this patient was subjected to multiple follow-up neuroimaging of standard sensitivity; henceforth, we suggest a functional disruption in the spinal tracts without any demonstrable structural damage.

The pathogenesis of SLE-associated myelopathy is considered mediated by (a) antiphospholipid antibodies (especially β2-glycoprotein)-mediated thromboembolic effects on spinal cord microcirculation, induction of antiaquaporin 4-antibodies following interactions of several lupus-autoantibodies with certain cord antigens, and direct cytotoxicity; (b) small-vessel vasculitis leading to cord ischemia and necrosis resulting in longitudinally extensive transverse myelitis; (c) change in blood–brain barrier; and (d) a result of several mechanisms (cord inflammation, venous hypertension, and cord ischemia) leading to hemodynamic compromise.^2-7,9,10^ Another important facet in the pathogenesis is the role of B cells and their subpopulations, which is well established in SLE. The hallmark of SLE is the production of autoantibodies by autoreactive B cells that trigger a hyperinflammatory response. Therefore, targeted B-cell suppression is a viable treatment option.^11^ B cells can also form tertiary lymphoid tissues that correlate strongly with immune complexes; thus, contributing to tissue inflammation and damage.^12^ Although this is a known phenomenon in kidney tissues, whether similar structures form in the spinal cord of patients with SLE requires further exploration. Indeed, B cells in the tertiary lymphoid organs may contribute to the inflammatory milieu by stimulating autoreactive T-cells.^13^

The history of recurrent mid-trimester pregnancy loss and antiphospholipid antibody positivity might indicate the potential role of anti-β2-glycoprotein-antibodies in this case. Simultaneous involvement of the peripheral neuroaxis besides central (myelopathy), particularly in the form of an acquired predominantly axonal poly(radiculo)neuropathy, has been seen in many cases of SLE-associated myelopathy, including the current case.^3,14,15^ Considering the classification of SLE-associated myelopathy proposed by Birnbaum et al.,^16^ our patient had features of predominant white-matter myelopathy overlapping with a few features of gray-matter myelopathy (hyperacute presentation, early bladder involvement, monophasic, high disease severity score, anti-β2-glycoprotein-antibodies positivity, and poor response to intravenous infusions of methylprednisolone/intravenous infusion of high-dose cyclophosphamide). As seen in subtle upper motor neuron type of bladder involvement and sensory tract involvements, early features of myelopathy can be easily misdiagnosed, especially when MRI reveals no signal changes.

Intravenous administration of methylprednisolone followed by cyclical intravenous high-dose infusion of cyclophosphamide alongside daily oral administration of hydroxychloroquine remains the mainstay of therapy. However, refractory cases are not rare and often require plasmapheresis or rituximab infusion.^2,7,17^ Since the patient could not bear the cost of performing plasmapheresis, we put her on rituximab infusion (supplied by the government, free of cost; after relevant precautionary work-ups for this drug). She responded well, and no recurrence occurred in 19 months of follow-up. Another treatment option is the FDA-approved belimumab (Benlysta), a fully humanized IgG1γ monoclonal antibody, which disrupts B-cell function by inhibition. Moreover, a recent randomized controlled trial showed that belimumab after rituximab therapy significantly reduced anti-dsDNA antibody levels and the risk of severe flares in patients with refractory SLE, pointing toward the utility of a combination therapy.^18^ However, as it was a government hospital and the patient could not afford the drug because of her lower socioeconomic status, we could not avail of this possible treatment option.

MRI-negative myeloneuropathy is a relatively new clinical entity, and much of its pathophysiological phenomenon is still unknown. This study’s ineffectiveness of global immunosuppressive treatments such as cyclophosphamide is intriguing. While cyclophosphamide is usually effective in managing neuropsychiatric manifestations of lupus, we should remember that recovery is not complete all the time. Although the initial nonresponsiveness with cyclophosphamide may indicate noninflammatory pathology, it is not easy to establish as such by extrapolating the experience of a single case. Secondary demyelination is also possible, but it lacks the tell-tale evidence of MR changes.

The surprising response with rituximab is also difficult to explain. However, the cumulative effects of initial cyclophosphamide therapy, potentiated later with the introduction of rituximab, could be an explanation. The effectiveness of rituximab usually starts 1 or 2 weeks after initiation of therapy (CD20 levels are used to monitor its effect). By extrapolating our experience of neuromyelitis optica treatment with rituximab,^19^ it can be said that clinical response often precedes CD20 suppression.

Another peculiar finding in this case was the bilateral lower motor neuron-type facial paresis, an uncommon peripheral neuraxial manifestation of neuropsychiatric syndromes of SLE.^20,21^ The pathophysiology of the lower motor neuron-type facial paresis in SLE might be the same as that of myelitis.^2-7,9,10^ Herein, thrombotic microvascular occlusion caused by antiphospholipid antibodies was considered as a possibility^20,21^; however, the rapid recovery following rituximab therapy does not support this hypothesis. Furthermore, spinal artery thrombosis-related myelitis was ruled out because of the absence of any characteristic pain and imaging abnormalities in this index case. Henceforth, thrombolytic treatment was not considered.

## Conclusion

Myelopathy and bilateral lower motor neuron-type facial paresis are rare and often underrecognized amid protean manifestations of SLE. MRI-negative myelopathy in SLE is an even rarer but documented condition that can be easily missed if not meticulously assessed during clinical history taking and examinations. Its pathomechanisms remain elusive, but antiphospholipid antibodies and other autoantigens might have a potential pathogenic role. Finally, timely identification and simultaneous institution of immunosuppressive therapeutics for such entity may prevent morbidity and mortality.

## Figures and Tables

**Table 1:** Common causes of acute- to subacute-onset myeloneuropathies and odds in this patient.

**Differential diagnoses**	**Odd(s) in this patient**

1. Nutritional deficiency	√ Normal serum B12, folic acid, vitamin E, and copper levels

2. Metabolic etiologies	√ No metabolic perturbation could be established throughout the disease

3. Toxin-related	√ No history of exposure to organophosphorus compounds and nitrous oxide

4. Drug-related	√ The patient was not on any drug that could be considered potentially neurotoxic

5. Infection-related	√ Relevant infectious diseases that can result in myeloradiculoneuropathy were ruled out with targeted serological and PCR-based tests

6. Tropical ataxic neuropathy	√ There was no history of cassava intake

7. Hashimoto’s	√ There were no cognitive impairments

myeloradiculoneuropathy	√ Euthyroid status

	√ The antithyroid peroxidase antibody was negative

	√ Electroencephalogram was normal

	√ MRI of the brain and spinal cord failed to delineate any abnormal signals

8. Paraneoplastic myeloneuropathy	√ No evidence of overt or unmanifested malignancy was found despite relevant screening

9. Adrenomyeloneuropathy	√ No evidence of associated adrenal leukodystrophy was found

10. Sjögren syndrome	√ Anti-SS-A and SS-B, Schirmer’s test, and salivary biopsy were negative

11. Neurosarcoidosis	√ Serum angiotensin-converting enzyme and calcium levels were normal

	√ High-resolution CT of the thorax was normal

12. Cauda equina and conus	√ MRI of the lumbosacral spine was noncontributory

medullaris syndromes	√ CSF study was noncorroborative

13. Friedreich’s ataxia	√ No features of inherited progressive cerebellopathy were found

